# Risk of radiation-induced pneumonitis after helical and static-port tomotherapy in lung cancer patients and experimental rats

**DOI:** 10.1186/s13014-015-0502-9

**Published:** 2015-09-17

**Authors:** Xianglan Zhang, You Keun Shin, Zhenlong Zheng, Lianhua Zhu, Ik Jae Lee

**Affiliations:** Department of Pathology, Yanbian University Hospital, Yanji, China; Oral Cancer Research Institute, Yonsei University College of Dentistry, Seoul, South Korea; Cancer Metastasis Research Center, Yonsei University College of Medicine, Seoul, South Korea; Department of Dermatology, Yanbian University Hospital, Yanji, China; Department of Radiation Oncology, Gangnam Severance Hospital, Yonsei University College of Medicine, 211 Eonju-ro, Gangnam-gu, Seoul 135-720 South Korea

## Abstract

**Background:**

Radiotherapy (RT) is one of the major non-operative treatment modalities for treating lung cancer. Tomotherapy is an advanced type of intensity-modulated radiotherapy (IMRT) in which radiation may be delivered in a helical fashion. However, unexpected pneumonitis may occur in patients treated with tomotherapy, especially in combination with chemotherapy, as a result of extensive low-dose radiation of large lung volumes. The aim of our study was to investigate the risk of radiation-induced pneumonitis after helical-mode and static-mode tomotherapy in patients with lung cancer and in an animal model.

**Method:**

A total of 63 patients with primary lung cancer who were treated with static or helical tomotherapy with or without concurrent chemoradiotherapy (CCRT) were analyzed. Additionally, rats with radiation-induced pulmonary toxicity, which was induced by the application of helical or static tomography with or without CCRT, were evaluated.

**Results:**

Helical-mode tomotherapy resulted in a significantly higher rate of late radiation pneumonitis in lung cancer patients than static-mode tomotherapy when evaluated by the Radiation Therapy Oncology Group (RTOG) and National Cancer Institute Common Terminology Criteria for Adverse Events (CTCAE) scoring system. In the animal model, helical tomotherapy alone induced significantly higher expression of interleukin (IL)-1α, IL-1β, IL-6, and transforming growth factor (TGF)-β in lung specimens, especially on the untreated side, compared to static tomotherapy alone. Additionally, rats treated with helical tomotherapy and CCRT demonstrated significantly higher expression of inflammatory cytokines compared to those treated with static tomotherapy and CCRT.

**Conclusion:**

Rat models treated with tomotherapy with or without CCRT could present similar patterns of pulmonary toxicity to those shown in lung cancer patients. The models can be used in further investigations of radiation induced pulmonary toxicity.

## Background

Radiotherapy (RT) is one of the major non-operative treatment modalities used for treating carcinomas of various organs, demonstrated by the fact that approximately half of all cancer patients have been shown to receive RT during their course of anti-cancer treatment [1, 2]. Ongoing investigations have improved the survival of cancer patients and minimized RT-related toxicities through the design of new RT modalities, including 3-dimensional conformal RT based on computed radiography imaging, image-guided RT, stereotactic body RT, and intensity-modulated (IM) RT [2]. Among these modalities, IMRT was developed to deliver irregularly shaped radiation doses as well as to save critical organs through the application of inverse planning software and computer-controlled intensity-modulation (2).

Tomotherapy (Accuray, Palo Alto, USA) is a form of IMRT which is integrated with an image guidance system, and helical tomotherapy delivers IMRT in a continuous helix for improved dose conformity to malignant tissues [3, 4]. Several oncologic diseases have been effectively and safely treated with helical tomotherapy, including prostate cancer, scalp angiosarcoma, hepatocellular carcinoma, breast cancer, and lung cancer [4–9]. Helical tomotherapy allows delivery of the radiation beam from any gantry angle as a result of continuous rotation around the patient, which results in the sparing of surrounding normal tissues from unwanted excessive radiation [3]. Generally, an increased fraction number has been considered to reduce the risk of radiation-induced high-grade toxicities [9]. However, unexpected pneumonitis may occur in patients treated with helical tomotherapy, especially in combination with chemotherapy, as a result of extensive low-dose radiation of large lung volumes [9, 10].

To avoid this risk of radiation-induced pneumonitis, a type of static tomotherapy using static gantry positions has been introduced, and a dosimetric study demonstrated optimaltarget volume coverage with adequate sparing of normal tissue with static tomotherapy [11]. In the current study, we compared the risk of radiation pneumonitis in patients with lung cancer who received either helical tomotherapy or static tomotherapy. In addition, we developed rat models of radiation pneumonitis, which were induced by helical tomography and static tomography with or without concurrent chemoradiotherapy (CCRT), respectively. Then, we evaluated the expression of radiation pneumonitis-associated cytokines, including transforming growth factor (TGF)-β, interleukin (IL)-1α, IL-1β, and IL-6, to determine the effects of radiation on surrounding normal lung tissues in each animal group.

## Materials and methods

### Patients

This study included 63 patients (43 males and 20 females; median age, 66 years; age range, 33–87 years) diagnosed with primary lung cancer who were treated with helical tomotherapy or static tomotherapy. Baseline characteristics of the participants are summarized in Table 1. The patients were pathologically diagnosed by surgical lung biopsy and staged according to the consensus recommendations of the International Association for the Study of Lung Cancer International Staging Committee [12].

**Table 1:** Patients’ baseline demographics and characteristics

		No. of patients (%)
Sex	Male	43 (68.3)
	Female	20 (31.7)
Age (years), median (range)		66 (33–87)
Smoking History	Yes	36 (57.1)
	No	27 (42.9)
Underlying COPD		3 (4.8)
Tumor location^a^	Right upper lobe	15 (23.8)
	Right middle lobe	6 (9.5)
	Right lower lobe	20 (31.7)
	Left upper lobe	19 (30.2)
	Left lower lobe	14 (22.2)
Histologic features	Adenocarcinoma	24 (38.1)
	Squamous cell carcinoma	16 (25.4)
	Small cell lung cancer	12 (19.0)
	Non-small cell lung cancer, NOS	9 (14.3)
	Bronchoalveolar carcinoma	1 (1.6)
	Large cell carcinoma	1 (1.6)
Aim of radiotherapy	Definitive	26 (41.3)
	Postoperative	1 (1.6)
	Salvage	19 (30.1)
	Palliative	17 (27.0)
Concurrent chemoradiotherapy	Yes	21 (33.3)
	No	42 (66.7)
PTV (cc), median (range)		144 (16–799)
Total lung V10 (%), median (range)		39 (5–93)
Total lung V20 (%), median (range)		16 (4–49)
Mode of tomotherapy	Static	12 (19.0)
	Helical	51 (81.0)

*COPD* chronic obstructive lung disease, *PTV* planning target volume, ^a^; 11 patients have multi-lobar lesions

All of the patients underwent helical tomotherapy (*N* = 51; 81.0 %) or static tomotherapy (*N* = 12; 19.0 %) for RT with definitive, salvage, or palliative treatment purposes. Tomotherapy was planned following the performance of chest computed tomography (CT), in which each patient was scanned from the mandible to the lower edge of the liver with 5-mm-thick slices. The gross tumor volume (GTV) included the primary tumor and involved regional lymphnodes, with a clinical target volume (CTV) of GTV plus a 0.6–0.8 cm margin and a planning target volume (PTV) of CTV plus a 0.5–1.0 cm margin. The patients usually received tomotherapy in five fractions per week at a radiation dose of 60–66 Gy in 2.0–2.2 Gy daily fractions.

Tomotherapy planning was performed using the TomoTherapy Hi-Art system version 4.0 (Accuray, Palo Alto, CA, USA). The planning system employed an inverse treatment planning process based on iterative least squares minimization of an objective function. The simultaneous integrated boost method was used in the planning for most patients. The importance factor placed constraints on the target and critical structures in relation to each other and determined the degree of weighting for achieving each prescription dose. For each target structure, a penalty factor was applied to the minimum and maximum dose values, and for organs at risk, it was used for the maximum dose. Each treatment plan was evaluated with a dose-volume histogram. In general, plans were considered acceptable if the PTV was covered by 95 % of the isodose curves, inhomogeneity of the PTV ranged from 95 % to 107 %, and doses to normal organs were limited in their tolerances. Dose tolerances for normal structures were as follows: total lung V20 < 40 %, mean lung dose (MLD) < 20 Gy, maximum dose to spinal cord < 45 Gy, heart V60 < 33 %, and esophagus V45 < 33 %.

Among the total of 63 patients, 21 (33.3 %) patients were treated with CCRT as either chemotherapy combined with static-mode tomotherapy (static-CCRT; *N* = 7) or helical-mode tomotherapy (helical-CCRT; *N* = 14). Chemotherapy was performed with a treatment regimen comprising cisplatin at a dose of 20–25 mg/m^2^/day on days 1–3 and docetaxel at 65–70 mg/m^2^/day on day 1, which was initiated simultaneously with tomotherapy. During the course of RT, 1–2 cycles of chemotherapy were concurrently administered, and after the complete of RT, additional 2–3 cycles of chemotherapy followed within 4 weeks.

The diagnosis of radiation pneumonitis was based on clinical symptoms, including dry cough, low-grade fever, shortness of breath, and chest pain, as well as the radiologic findings on chest X-ray and CT scan read by at least two radiologists. Most cases of acute radiation pneumonitis occur between 6 and 12 weeks after the conclusion of RT, whereas late radiation pneumonitis generally presents after 6 months [13]. The risk of radiation pneumonitis was evaluated by the grading systems of both the Radiation Therapy Oncology Group (RTOG) and the National Cancer Institute Common Terminology Criteria for Adverse Events (CTCAE), version 3.0 [14]. Our experimental protocols were approved by the Institutional Review Board of Severance Hospital at Yonsei University College of Medicine in Seoul, Korea.

### Animal model and treatment protocol

Five-week-old Sprague–Dawley rats (*N* = 15, body weights of 300–400 g) were divided into five groups; Group 1: controls (*N* = 3), Group 2: rats treated with static tomotherapy alone (*N* = 3), Group 3: helical tomotherapy alone (*N* = 3), Group 4: static-CCRT (*N* = 3), and Group 5: helical-CCRT (*N* = 3). Chemotherapy was performed by intraperitoneal injection of gemcitabine (2′,2’-difluoro-2’-deoxycytidine) (Eli Lilly and Company, Indianapolis, IN, USA) at a dose of 5 mg/kg body weight as described previously [15]. RT was planned by CT simulation and a dose of 12 Gy was delivered to the right lobe of the lung under anesthesia with phenobarbital at a dose of 30 mg/kg (Sigma-Aldrich Co. LLC, St. Louis, MO, USA). The left lung was protected by a constraint set to minimize radiation exposure in both helical and static tomotherapy. Target regions were irradiated with a margin of at least 2 mm in width from the center in order to prevent radiation esophagitis or myelitis. Other regions were protected from irradiation with a multileaf collimator. The animal studies were performed according to experimental protocols that were approved by the Committee for the Care and Use of Laboratory Animals of Severance Hospital. The experiments were repeated twice. Similar patterns were found between the first experiments and repeated experiments.

### Enzyme-linked immunosorbent assay

Peripheral blood levels of IL-1α (Abcam, Cambridge, UK) and IL-1β (Abcam) were detected using enzyme-linked immunosorbent assay (ELISA) kits according to the manufacturers’ instructions. Peripheral blood samples were obtained from tail veins of experimental rats for 1, 2, and 3 weeks after tomotherapy and CCRT. For ELISA, 2 mL of peripheral blood was collected from each experimental rat followed by isolation of serum. Samples with inadequate serum quantity or quality due to hemolysis were excluded from biochemical measurements. Additionally, serum samples were measured in triplicate and those showing more than three-time variation from the mean value were excluded from the study.

### Histological and immunohistochemical analysis

Tissues from the right and left lungs of experimental animals were obtained at 3 weeks after tomotherapy or CCRT, fixed in 10 % buffered formalin, and embedded in paraffin. Serial tissue sections of 4-μm thickness were prepared for histological analysis with hematoxylin and eosin (H&E) stain. Additionally, immunohistochemical stains were performed with prepared tissue sections. Briefly, the sections were deparaffinized with xylene and then rehydrated in graded ethanol. Antigen retrieval was achieved by pressure-cooking using Antigen Retrieval Solution (Dako, Carpinteria, CA, USA). Then, endogenous peroxidase activity was blocked with Endogenous Enzyme Block (Dako). The sections were incubated with primary antibodies, including anti-IL-1α antibody (Abcam) and rabbit anti-IL-1β polyclonal antibody (Santa Cruz Biotechnology, Santa Cruz, CA, USA) at a dilution of 1:100, rabbit anti-IL-6 polyclonal antibody (Abcam) at 1:50, and mouse anti-TGF-β monoclonal antibody (Abcam) at 1:25 for 1 h at room temperature. Real™EnVision™ HRP Rabbit/Mouse detection system (Dako) was used as a secondary antibody, and the sections were then counterstained with hematoxylin.

Rabbit anti-IgG antibody (R&D Systems GmbH, Wiesbaden, Germany) or mouse anti-IgG antibody (DakoCytomation GmbH, Hamburg, Germany) were used as isotype-matched control antibodies at concentrations identical to those of the primary antibodies. The staining intensity of the tumor cells was scored as 0 (negative), 1 (light brown), 2 (brown), and 3 (dark brown). The final score was calculated as (0 × % negative cells) + (1 × % light brown-stained cells) + (2 × % brown-stained cells) + (3 × % dark brown-stained cells) according to a weighted histoscore method [16].

### Statistical analysis

Expression of IL-1α, IL-1β, IL-6, and TGF-β in each group was compared using the Mann–Whitney test. Statistical analyses were carried out using SPSS software (version 14.0; SPSS Inc., Chicago, IL, USA) and the differences were considered significant at a value of *p* less than 0.05.

## Results

### Frequency of pneumonitis in lung cancer patients treated with tomotherapy

The frequency of radiation pneumonitis was comparatively investigated in lung cancer patients who received tomotherapy using either the helical or the static mode. A total of 63 patients were evaluated for acute radiation pneumonitis, and 54 patients were then investigated for late radiation pneumonitis as 9 patients expired over the course of follow-up. The frequency of CCRT in the group of patients treated with helical-mode tomotherapy was higher than in those treated with the static mode (*p* = 0.04). However, both acute and late pneumonitis of ≥ grade 2 according to the RTOG and CTCAE scoring systems occurred more frequently in the group of patients treated with helical-mode tomotherapy than in those treated with the static mode over the course of RT (Table 2). Additionally, statistical analyses revealed that helical-mode tomotherapy resulted in a significantly higher rate of late radiation pneumonitis than static-mode tomotherapy when evaluated by the RTOG and CTCAE scoring system (*p* = 0.001, and 0.01).

**Table 2:** A comparison of patient characteristics and radiation pneumonitis in lung cancer patients treated with tomotherapy according to the delivery mode of radiation beams

		Mode of tomotherapy		*P*-value
		Static	Helical	
Smoking History	Yes	12/12 (83.3 %)	26/51 (51.0 %)	0.04
	No	2/12 (16.7 %)	25/51 (49.0 %)	
PTV (cc)	Median (range)	158.50 (57–799)	144.00 (16–789)	*NS*
Total lung V10 (%)	median (range)	32.00 (11–70)	44.00 (5–98)	*NS*
Total lung V20 (%)	median (range)	16.00 (5–51)	16.00 (4–53)	*NS*
CCRTx	Yes	7/12 (58.3 %)	14/51 (27.5 %)	0.04
	No	5/12 (41.7 %)	37/51 (72.5 %)	
Radiation pneumonitis scoring system				
RTOG	Acute	3/ 12 (25.0 %)	15/51 (29.4 %)	*NS*
	Late	1/ 12 (8.3 %)	27/42 (64.3 %)	0.001
CTCAE	Acute	3/ 12 (25.0 %)	20/51 (39.2 %)	*NS*
	Late	1/ 12 (8.3 %)	20/42 (47.6 %)	0.01

*CCRTx* concurrent chemoradiotherapy, *RTOG* Radiation Therapy Oncology Group, *CTCAE* the National Cancer Institute Common Terminology Criteria for Adverse Events, version 3.0, *NS* not significant

Of the radiotherapeutic parameters, late grade 2 or worse radiation pneumonitis by CTCAE score was observed in 28.9 % (11 of 38 patients) with a total lung V10 Gy of < 50 % and in 62.5 % (10 of 16 patients) with a total lung V20 Gy of ≥ 60 % (*p =* 0.02). Acute grade 2 or worse radiation pneumonitis by CTCAE scoring system was observed in 10 (26.3 %) of 38 patients with a total lung V20 Gy of < 20 % and in 13 (52.0 %) of 25 patients with a total lung V20 Gy of ≥ 60 % (*p =* 0.04).

### Plasma levels of IL-1α and IL-1β according to the experimental rat groups

**Fig. 1:**
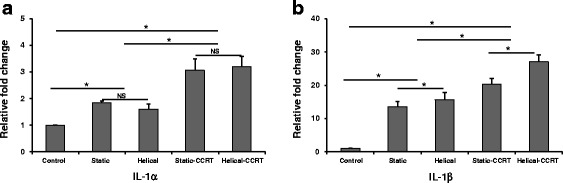
Plasma levels of **a** IL-1α and **b** IL-1β according to the experimental rat groups. There were significant differences among the groups at 3 weeks after tomotherapy or CCRT. ^*^ *p* <0.05; NS, not significant; IL, interleukin; CCRT, concurrent chemoradiotherapy

Plasma levels of IL-1α and IL-1β in each experimental rat group were comparatively evaluated at 1 week, 2 weeks, and 3 weeks after tomotherapy or CCRT. No significant differences in the levels of expression of IL-1α and IL-1β were found among the groups at 1 week or 2 weeks. However, there were significant differences among the groups at 3 weeks after tomotherapy or CCRT; expression of IL-1α was 1.8-fold (Group 2; *p* = 0.037), 1.6-fold (Group 3; *p* = 0.037), 3.1-fold (Group 4; *p* = 0.037), and 3.2-fold (Group 5; *p* =0.037) higher compared to the control group (Group 1) (Fig. 1a), and expression of IL-1β was also 13.5-fold (Group 2; *p* =0.037), 16.6-fold (Group 3; *p* = 0.037), 20.3-fold (Group 4; *p* =0.037), and 27.1-fold (Group 5; *p* = 0.037) higher (Fig. 1b).

The plasma levels of IL-1α at 3 weeks after the treatments were significantly higher in the static-CCRT group (Group 4) compared to the groups that received either static tomography alone (Group 2; *p* = 0.047) or helical tomotherapy alone (Group 3; *p* =0.047), as well as in the helical-CCRT group (Group 5) compared to the groups that received either static tomography alone (*p* =0.047) or helical tomotherapy alone (*p* =0.047). However, the plasma IL-1α levels were not significantly different between the groups that received static tomotherapy alone and helical tomotherapy alone or between the static-CCRT group and the helical-CCRT group.

Additionally, the plasma levels of IL-1β at 3 weeks after the treatments were significantly higher in the static-CCRT group compared to the groups that received either static tomotherapy alone (*p* = 0.047) or helical tomography alone (*p* = 0.047), as well as in the helical-CCRT group compared to the groups that received either static tomotherapy alone (*p* = 0.047) or helical tomography alone (*p* = 0.047). Moreover, IL-1β expression was significantly increased in the helical tomotherapy alone group compared to the group that received static tomotherapy alone (*p* = 0.047), as well as in the helical-CCRT group compared to the static-CCRT group (*p* =0.047).

### Tissue expression of IL-1α in the animal model

**Fig. 2:**
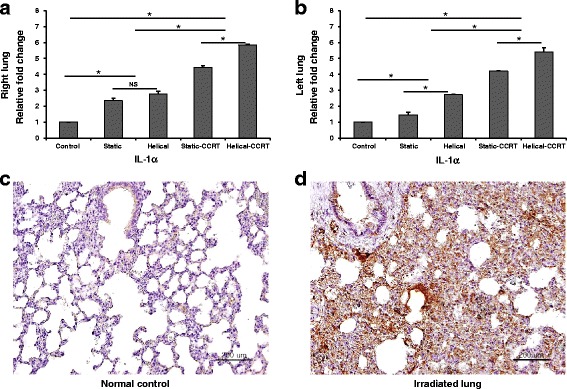
Tissue expression of IL-1α. **a** Right lung samples demonstrated higher tissue IL-1α expression compared to the untreated control samples. **b** Left lung samples revealed upregulated tissue IL-1α expression. Immunohistochemical staining with anti-IL-1α antibody of **c** normal control and **d** right lung sample treated with helical tomotherapy with CCRT. ^*^ *p* <0.05; NS, not significant; IL, interleukin; CCRT, concurrent chemoradiotherapy; bars = 200 μm

Lung specimens were obtained from the experimental rats in each group at 3 weeks after tomotherapy or CCRT, and tissue expression of IL-1α, IL-1β, IL-6, and TGF-β was evaluated using immunohistochemical staining and weighted histoscoring. Right lung samples from the treatment groups demonstrated 2.4-fold (Group 2, *p* = 0.037), 2.8-fold (Group 3, *p* = 0.037), 4.4-fold (Group 4, *p* = 0.037), and 5.8-fold (Group 5, *p* = 0.037) higher tissue expression of IL-1α compared to the untreated right lung samples (Group 1) (Fig. 2a, c, d). Additionally, left lung samples from the treatment groups showed 1.5-fold (Group 2, *p* = 0.037), 2.7-fold (Group 3, *p* = 0.037), 4.2-fold (Group 4, *p* = 0.037), and 5.4-fold (Group 5, *p* = 0.037) higher tissue expression of IL-1α compared to the untreated left lung specimens of Group 1 (Fig. 2b).

Both right and left lung samples obtained from the rats in groups treated with CCRT were found to have higher IL-1α expression compared to the groups treated with RT alone (right lung, *p* = 0.004; left lung, *p* = 0.004). Additionally, rats treated with helical tomotherapy alone demonstrated significantly higher levels of IL-1α compared to those treated with static tomotherapy alone (right lung, *p* = 0.047; left lung, *p* = 0.047). Helical-CCRT in rats resulted in significantly higher IL-1α expression compared to static-CCRT in both the right and left lungs (*p* = 0.047 and *p* = 0.047, respectively).

### Tissue expression of IL-1β in the animal model

**Fig. 3:**
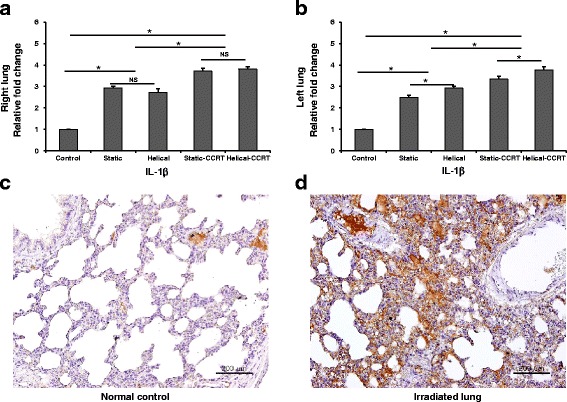
Tissue expression of IL-1β. **a** Right lung samples demonstrated higher tissue IL-1β expression compared to the untreated control samples. **b** Left lung samples revealed upregulated tissue IL-1β expression. Immunohistochemical staining with anti-IL-1β antibody of **c** normal control and **d** right lung sample treated with helical tomotherapy with CCRT. ^*^ *p* <0.05; NS, not significant; IL, interleukin; CCRT, concurrent chemoradiotherapy; bars = 200 μm

Significantly upregulated tissue expression of IL-1β was found in both right and left lung samples obtained from rats in Group 2 (2.9-fold, *p* = 0.037; 2.5-fold, *p* = 0.037, respectively), Group 3 (2.7-fold, *p* = 0.037; 2.9-fold, *p* = 0.037, respectively), Group 4 (3.7-fold, *p* = 0.037; 3.3-fold, *p* = 0.037, respectively), and Group 5 (3.8-fold, *p* = 0.037; 3.7-fold, *p* = 0.037, respectively) when compared to the untreated control group (Group 1) (Fig. 3a–d). Both right and left lung samples obtained from the rats in groups treated with CCRT were found to have higher IL-1β expression compared to the groups treated with RT alone (right lung, *p* = 0.004; left lung, *p* = 0.004). Rats treated with helical tomotherapy alone demonstrated significantly higher tissue expression of IL-1β compared to those treated with static tomotherapy alone in the left lung (*p* = 0.047), but not in the right lung (*p* > 0.05). Additionally, rats treated with helical-CCRT showed significantly higher IL-1β levels compared to those treated with static-CCRT (right lung, *p* > 0.05; left lung, *p* = 0.047).

### Tissue expression of IL-6 in the animal model

**Fig. 4:**
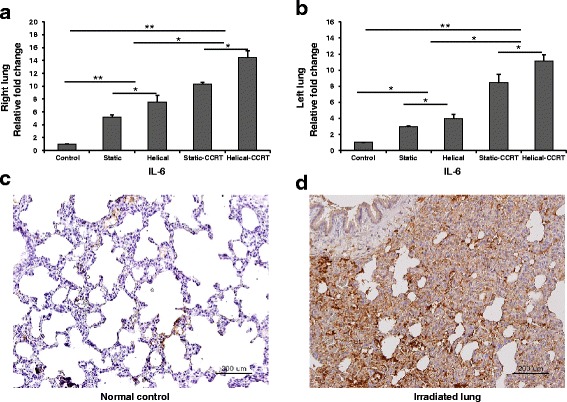
Tissue expression of IL-6. **a** Right lung samples demonstrated higher tissue IL-6 expression compared to the untreated control samples. **b** Left lung samples revealed upregulated tissue IL-6 expression. Immunohistochemical staining with anti-IL-6 antibody of **c** normal control and **d** right lung sample treated with helical tomotherapy with CCRT. ^*^ *p* <0.05; ^**^ *p* <0.01; IL, interleukin; CCRT, concurrent chemoradiotherapy; bars = 200 μm

Significantly upregulated tissue expression of IL-6 was found in both right and left lung samples obtained from rats in Group 2 (5.2-fold, *p* = 0.037; 3-fold, *p* = 0.037, respectively), Group 3 (7.5-fold, *p* = 0.037; 4-fold, *p* = 0.025, respectively), Group 4 (10-fold, *p* = 0.034; 8.5-fold, *p* = 0.037, respectively), and Group 5 (14.5-fold, *p* = 0.037; 11-fold, *p* = 0.037, respectively) when compared to the untreated control group (Fig. 4a–d). Both right and left lung samples obtained from the CCRT-treated rats were found to have higher IL-6 levels compared to the groups treated with RT alone (right lung, *P* = 0.004; left lung, *p* = 0.004). Rats treated with helical tomotherapy alone demonstrated significantly higher IL-6 expression compared to those treated with static tomotherapy alone (right lung, *p* = 0.047; left lung, *p* = 0.037). Additionally, helical-CCRT treatment resulted in remarkably higher tissue IL-6 expression compared to static-CCRT treatment (right lung, *p* = 0.046; left lung, *p* = 0.047).

### Tissue expression of TGF-β in the animal model

**Fig. 5:**
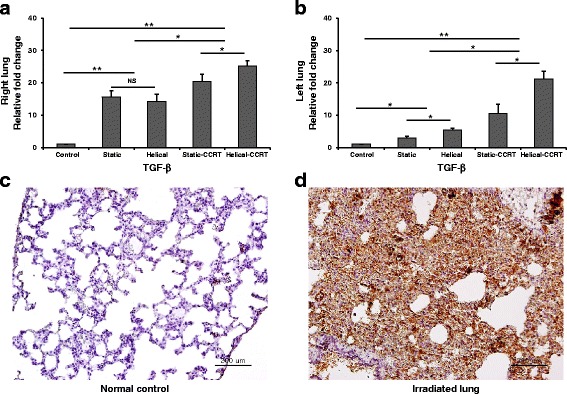
Tissue expression of TGF-β. **a** Right lung samples demonstrated higher tissue TGF-β expression compared to the untreated control samples. **b** Left lung samples revealed upregulated tissue TGF-β expression. Immunohistochemical staining with anti-TGF-β antibody of **c** normal control and **d** right lung sample treated with helical tomotherapy with CCRT. ^*^ *p* <0.05; ^**^ *p* <0.01; NS, not significant; TGF, transforming growth factor; CCRT, concurrent chemoradiotherapy; bars = 200 μm

Right lung samples from the treatment groups demonstrated 15.7-fold (Group 2, *p* = 0.037), 14.2-fold (Group 3, *p* = 0.037), 20.3-fold (Group 4, *p* = 0.037), and 25.3-fold (Group 5, *p* = 0.037) higher tissue expression of TGF-β compared to the right lung tissues of untreated controls (Fig. 5a, c, and d). Additionally, left lung samples from the treatment groups showed 2.5-fold (Group 2, *p* = 0.037), 5.5-fold (Group 3, *p* =0.037), 10.5-fold (Group 4, *p* = 0.037), and 21.2-fold (Group 5, *p* = 0.037) higher tissue TGF-β expression compared to the controls (Fig. 5b).

Both right and left lung samples obtained from the rats in groups treated with CCRT were found to have remarkably higher TGF-β expression compared to the RT alone groups (right lung, *p* = 0.006; left lung, *p* = 0.004). Helical tomotherapy alone induced significantly higher TGF-β expression in lung specimens compared to static tomotherapy alone (right lung, *p* > 0.05; left lung, *p* = 0.047). Additionally, rats treated with helical-CCRT demonstrated significantly higher TGF-β expression compared to those treated with static-CCRT (right lung, *p* = 0.047; left lung, *p* =0.047).

## Discussion

Radiation-induced pneumonitis occurs in various cancerous conditions, including small cell or non-small cell carcinoma of the lung, esophageal cancer, breast cancer, and malignant lymphoma, as a complication of thoracic RT [17]. In patients with lung cancers, the risk of radiation pneumonitis is significantly correlated with the dose and volume factors of RT [18]. When the total lung volume receiving 20 Gy is less than 22 %, radiation pneumonitis rarely occurs, whereas an 8 % risk of grade 2 radiation pneumonitis can be encountered in cases with 22–31 % of the lung volume receiving 20 Gy [18]. Additionally, a dose-volume histogram analysis study demonstrated that the dosimetric factors of normal tissue complication probability, mean dose, and total lung volume receiving ≥30 Gy were significantly associated with radiation-induced pneumonitis [19].

The lung volume receiving high doses has been reduced by the advent of IMRT, however, the risk of acute or late pulmonary toxicity should be considered as the volume receiving low doses has been increased [17, 20, 21]. Helical-mode tomotherapy delivers low-dose exposure of almost the entire lung volume at target area level. Several authors reported helical tomotherapy is safe and not associated with a specific pattern of lung injury [22]. Donato et al. assessed the toxicities of hypofractionated radiation course delivered with helical tomotherapy, and they reported acute grade 3 treatment-related pneumonitis was detected in 10 % of patients. These findings not increased the risk of radiation pneumonitis when compared with other observed studies.

Song et al. assessed clinical outcomes in non-small-cell lung cancer patients treated with chemotherapy and helical tomotherapy, and reported four patients died of treatment-related death after helical-mode radiotherapy [23]. They suggested that helical tomotherapy has high rate of fatal pulmonary complications. Kim et al. reported low dose V5 of ipsilateral and contralateral lung volume were also significant predictive factors for radiation pneumonitis after helical tomotherapy [24]. They recommended normal lung of V5, V10 and V15 should be kept as low as possible. To reduce the chance of low-dose irradiation to large areas of normal tissue, a type of static tomotherapy, which uses static gantry positions, has been performed in combination with multileaf collimator modulation and couch translation [11, 25]. The planning time of IMRT can be reduced in static-mode tomotherapy by using specified beam angles compared with the helical-mode [11, 25]. Additionally, the beam-on time is lesser in the static-mode than in the helical mode and conventional 3-dimensional conformal RT [11]. Therefore, optimal target volume coverage with adequate sparing of normal tissue (e.g.,lung volume) can be achieved by using static-mode tomotherapy [25].

In this study, we compared the risk of radiation pneumonitis between helical tomotherapy and static tomotherapy in lung cancer patients. Although, the frequency of CCRTx in the group of patients treated with helical-mode tomotherapy was higher than in those treated with the static mode (*p* = 0.04), they found that helical-mode tomotherapy resulted in a significantly higher rate of late radiation pneumonitis than static-mode tomotherapy.

Additionally, we developed rat models of radiation pneumonitis, which were induced by helical tomography and static tomography. Our evaluation of tissue samples from treated lung found that the expression of radiation-induced inflammatory cytokines, including TGF-β, IL-1α, IL-1β, and IL-6, was similarly increased in groups treated with helical-mode and static-mode tomotherapy. However, when comparing samples from the opposite side of the lung, we found that radiation-induced inflammatory cytokines were significantly increased in rat groups treated with helical-mode tomotherapy compared to those treated with static-mode tomotherapy.

The risk of radiation-induced pulmonary toxicity is remarkably increased by the concomitant systemic therapy of CCRT [17]. However, dose-volume recommendations which are specific for CCRT have not been fully established, with the exception of the recommendations of total lung volume receiving 20 Gy and 30 Gy [17–19]. In our study, we found that animal models also exhibited significantly remarkable pulmonary toxicity on the treated side of the lungs in the groups that received CCRT compared to those treated with helical-mode and static-mode tomotherapy alone. Moreover, when comparing the opposite side of the lung, we found that radiation-induced inflammatory cytokines were increased in the rat group treated with helical-CCRT compared to the static-CCRT group.

## Conclusion

In the present study, we found that rat models treated with helical-mode or static-mode tomotherapy with or without CCRT could present similar patterns of pulmonary toxicity to those shown in lung cancer patients. Nevertheless, further studies are needed to establish an appropriate animal model of radiation-induced pulmonary toxicity to exactly reflect the radiation-induced human tissue response. Additionally, we suggest that our study can be used as a basic reference for developing an animal model of radiation-induced pulmonary toxicity.

## Abbreviations

RT: Radiotherapy
IMRT: Intensity-modulated radiotherapy
CCRT: Concurrent chemoradiotherapy
RTOG: Radiation Therapy Oncology Group
CTCAE: Cancer Institute Common Terminology Criteria for Adverse Events
IL: Interleukin
TGF: Transforming growth factor
CT: Computed tomography
GTV: Gross tumor volume
CTV: Clinical target volume
PTV: Planning target volume
MLD: Mean lung dose
ELISA: Enzyme-linked immunosorbent assay
H&E: Hematoxylin and eosin
